# Automatic Pulmonary Nodule Detection Applying Deep Learning or Machine Learning Algorithms to the LIDC-IDRI Database: A Systematic Review

**DOI:** 10.3390/diagnostics9010029

**Published:** 2019-03-07

**Authors:** Lea Marie Pehrson, Michael Bachmann Nielsen, Carsten Ammitzbøl Lauridsen

**Affiliations:** 1 Department of Diagnostic Radiology, Copenhagen University Hospital, Rigshospitalet, 2100 Copenhagen, Denmark; Mbn@dadlnet.dk (M.B.N.); Cala@kp.dk (C.A.L.); 2 Department of Technology, Faculty of Health and Technology, University College Copenhagen, 2200 Copenhagen, Denmark

**Keywords:** deep learning, machine learning, nodule detection

## Abstract

The aim of this study was to provide an overview of the literature available on machine learning (ML) algorithms applied to the Lung Image Database Consortium Image Collection (LIDC-IDRI) database as a tool for the optimization of detecting lung nodules in thoracic CT scans. This systematic review was compiled according to Preferred Reporting Items for Systematic Reviews and Meta-Analyses (PRISMA) guidelines. Only original research articles concerning algorithms applied to the LIDC-IDRI database were included. The initial search yielded 1972 publications after removing duplicates, and 41 of these articles were included in this study. The articles were divided into two subcategories describing their overall architecture. The majority of feature-based algorithms achieved an accuracy >90% compared to the deep learning (DL) algorithms that achieved an accuracy in the range of 82.2%–97.6%. In conclusion, ML and DL algorithms are able to detect lung nodules with a high level of accuracy, sensitivity, and specificity using ML, when applied to an annotated archive of CT scans of the lung. However, there is no consensus on the method applied to determine the efficiency of ML algorithms.

## 1. Introduction

Machine learning (ML) and deep learning (DL) are becoming established disciplines in the broad field of applying artificial intelligence in analyzing and utilizing patterns in datasets. As the complexity and shear amount of data increase, applying these patterns for the benefit of, e.g., clinical decision making, becomes increasingly nontrivial [1]. Extraordinary advancements in areas of technology such as high-performance computing have made it possible to attempt solving these problems algorithmically. The purpose of various ML and DL algorithms may be to improve quality, consistency, and/or capacity of data interpretation in diagnostics, thus improving diagnostics and treatment decisions to the benefit of clinical outcomes. Considering the implications this may have for the practice of medicine and healthcare, it is important to engage in this area of research from many perspectives. ML is already being applied to the practice of radiology, and the systems being developed today are showing to be robust in real-world conditions [2]. Several reviews have been published reviewing these techniques [3]. The Cancer Imaging Archive (TCIA) has the largest annotated public database, known as the Lung Image Database Consortium Image Collection (LIDC-IDRI), containing 1018 cases [4]. Since 2014, there have not been any systematic reviews published concerning the application of ML for the optimization of detecting pulmonary nodules in CT scans from the LIDC-IDRI database. The database is created with the intent to further the development of the training and evaluation of computer-assisted diagnostic (CAD) methods for lung cancer detection and diagnosis. The aim of this systematic review is, therefore, to provide an overview of the published literature, in order to evaluate the algorithm’s ability to detect lung nodules in CT scans released by the LIDC-IDRI database.

## 2. Materials and Methods

The eligibility criteria and analysis in this review were performed according to the PRISMA guidelines 2009 (Preferred Reporting Items for Systematic Reviews and Meta-Analyses) [5]. The literature search was completed on 22 November 2018. The literary search was performed in PubMed, Web of Science, Scopus, The Institute of Electrical and Electronics Engineers, Inc. (IEEE), and the association for computing machinery library database. This was done to identify publications that apply ML or DL algorithms to the LIDC-IDRI database for optimizing lung nodule detection. The selection criteria for the publications included are articles written in English and published since the 1 January 2014. To perform the search, the following expressions were used:

Search string number 1: (“3D” OR “3-dimensional” OR “three-dimensional”) AND (“detection” OR “segmentation” OR “cad” OR “cade”) AND (“lung” OR “lungs” OR “pulmonary” OR “chest”) AND (“nodule” OR “nodules” OR “cancer” OR “tumor” OR “tumors”).

Search string number 2: (“deep learning” OR “machine learning”) AND (“detection” OR “segmentation” OR “feature” OR “feature extraction” OR “features” OR “classification”) AND (“The lung image database consortium” OR “LIDC”) AND (“Lung nodule” OR “nodule detection”).

**Figure 1:**
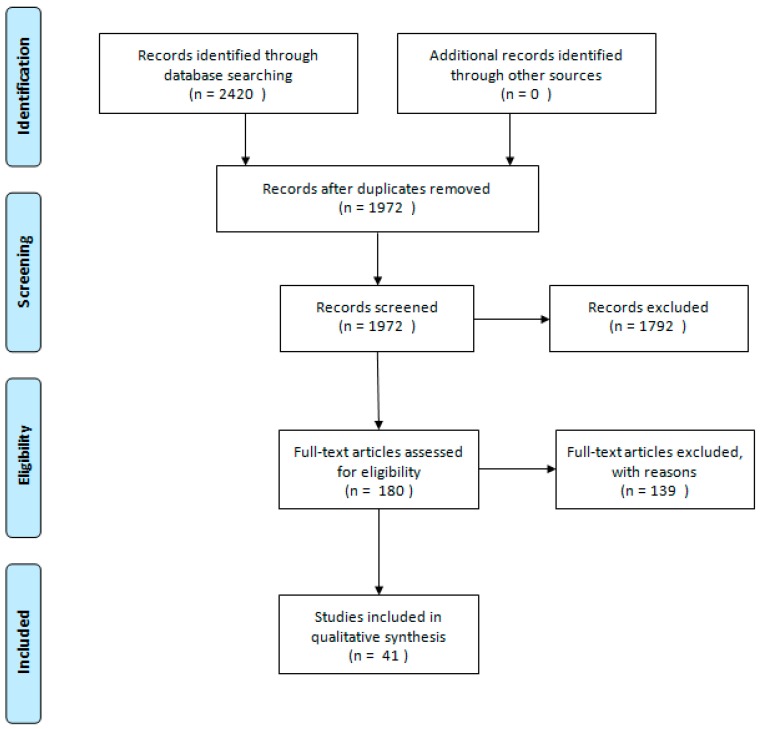
Preferred Reporting Items for Systematic Reviews and Meta-Analyses (PRISMA) flowchart of the literature search and study selection.

The first search expression is inspired by a previous review from 2014 [3]. The second search is presented by the author, and has been applied to the databases to identify publications that apply the latest technology related to lung nodule detection. By applying two search strings, we were able to include a larger number of articles. After removal of duplicates, all studies included in the search result were screened by title and abstract by two authors (L.M.P. and C.A.L.). Original research articles concerning algorithms applied to the LIDC-IDRI database were included. The LIDC-IDRI is the largest annotated database on thoracic CT scans [4]. The articles were subsequently retrieved and read by the same authors. Consensus was reached through discussion. All reference lists of the included articles were manually searched for further references. The articles were divided into two groups based on the type of algorithm presented in the articles. Table 1 presents 19 articles that apply a feature-based learning algorithm. Table 2 presents 22 articles that apply DL algorithms in order to detect lung nodules. The inclusion criteria for articles included all apply a type of ML algorithm to the LIDC-IDRI database and present results that showcase the algorithm’s accuracy, sensitivity, and specificity, and ability to obtain area under the curve (AUC) results; and were published from 1 January 2014. The articles that did not present at least one of these criteria and applied the LIDC-IDRI database were excluded. After removal of duplicates, the initial search yielded 1972 publications, 1792 of which were excluded. A total of 180 full-text articles were assessed for eligibility and 139 of these articles were excluded because of lacking data requirements, leaving 41 articles that were included in this systematic review. The study selection is summarized in Figure 1.

The LIDC-IDRI is the largest publicly available annotated CT database. It consists of 7371 lesions marked as a nodule by at least one radiologist. Of these lesions, 2669 were at least 3 mm or larger, and annotated by, at minimum, one radiologist. Out of the 2669 lesions, 928 (34.7%) received the same mark by four radiologists. The 2669 lesions are outlined and subjective nodule characteristics are all annotated. The LIDC-IDRI required the four radiologists to independently review each scan and mark lesions identified with respect to specific criteria described in Armato et al. 2014 [4].

The following is an acknowledged definition of ML: The algorithm is applied to a dataset, in this case, the LIDC-IDRI database. The annotations of nodules and the estimated malignancy of the nodule in the training data are learned by the algorithm. The knowledge obtained from the training set allows the algorithm to learn to make predictions. The prediction, in this case, could be whether there is a nodule located on the slice or whether the nodule is benign or malignant. Altering the features that are given can lead to an improvement in diagnosis. If the algorithm is able to optimize the parameters, it is considered to be learning the task. There are several differences between DL algorithms and algorithms based on hand-engineered features: the structure of the algorithm differs—the DL algorithm usually consists of several hidden layers; the two approaches require different input information; the algorithms based on hand-engineered features require proper segmentation of the nodule from a radiologist, or a segmentation algorithm and further quantitative image feature extraction; and the DL approach does not need the same elaborative segmentation process as the algorithms are able to make predictions from one marked point per nodule [6]. The data extracted from the articles are the results presented by the authors. These results showcase the algorithm’s ability to achieve the highest accuracy, sensitivity, and specificity, and derive AUC values.

## 3. Results

### 3.1. Algorithms Applying a Feature-Based Framework

Table 1 shows the 19 studies that have applied a feature-based framework. This table correspondingly showcases the best results the studies achieved using a specific algorithm. In the table, the studies marked with a star (“*”) presented several types of alterations to the algorithm, producing different results. These results are not presented in the table. Furthermore, if the data were unable to be obtained from the publication, this is stated as Not Available (NA).

### 3.2. Support Vector Machine (Six Studies)

Eight out of 19 studies proposed an algorithm applying a type of Support-vector machine (SVM) classifier [7,8,10,12,13,20,21,24]. These studies achieved some of the best results with regards to accuracy, sensitivity, specificity, and AUC, and all applied an SVM classifier and a type of feature extraction with focus on shape, intensity, or texture. The algorithms that applied an SVM classifier reached a range of accuracy of 68.4%–99.0%, sensitivity of 55.0%–98.6%, specificity of 87.5%–98.2%, and an AUC of 0.905–0.998.

The table displays 12 studies marked with a star [7,8,10,12,13,14,16,20,21,22,23,24]. These studies applied several alternative combinations of features, classifiers, or validation methods. Eight of these algorithms achieved the best results while applying an SVM type of classifier, and the best of all the algorithms, except one, reached an accuracy range of 96.7%–99.0% [7,8,10,12,20,21,24].

### 3.3. Other Classifiers (Six Studies)

Gong et al. [14] tested the algorithms on an Fisher linear discriminant analysis (FLDA) and naïve Bayes classifier. The FLDA classifier obtained the highest AUC and sensitivity compared to the naïve Bayes classifier. Gupta et al. [15] applied a stacked autoencoder to acquire an unsupervised neural network. A softmax layer was stacked with the autoencoder to perform the classification. The softmax classifier was used to solve binary classification problems. Hancock et al. [16] tested linear and nonlinear classifiers combined with either features included or excluded. The best performing algorithm was the nonlinear classifier, including the diameter and volume features. The nonlinear classifier with features excluded outperformed both the linear classifiers.

Lu et al. [19] presented an algorithm consisting of a hybrid method. The method integrated existing and often-applied algorithms. Taşcı et al. [22] presented an algorithm for detection of juxtrapleural nodules. The algorithm was initially tested on ten different classifiers. The best performing classifier out of the ten tested was the generalized linear model regression (GLMR) classifier, which utilized 22 out of 33 features and achieved an accuracy of 92.9%.

Liu et al. [18] presented a multilayer, fully connected network and consists of one input layer, one hidden layer, and one linear output layer. Wang et al. proposed a new classifier, utilizing semi-supervised extreme learning machine (SS-ELM). The proposed method achieved better results compared to applying an extreme learning machine (ELM), SVM, probabilistic neural network (PNN), and multilayer perceptron (MLP) classifier.

Bai et al. [9] combined a model-based local shape analysis and data-driven local contextual feature learning to improve detection in low dose CT. The algorithm applied a random forest trained to learn and combine a subset of these primitives into discriminative orientation invariant contextual features and classify nodule candidates. By applying this method, the algorithm reached a sensitivity of 80%. Liu et al. [18] proposed an ANN algorithm trained on the LIDC-IDRI database. The algorithm applied 3D geometric and statistical features to constitute a voting method. While applying this method the algorithm reached a sensitivity of 89.4%. El Regaily [11] applied a simple rule classifier and achieved a total accuracy of 70.53%. These results were satisfactory, taking into consideration the classifier applied on the initial first step of the classification. The author proposed to apply a SVM classifier in order to raise the accuracy and reduce the amounts of FP.

Jaffar et al. [17] achieved the highest sensitivity, specificity, and AUC. This was done while applying a random forest classifier. The study proposed a novel ensemble shape gradient features (NESGF) descriptor for pulmonary nodule classification using the histogram of oriented surface normal vectors and multi-coordinate histogram of gradient descriptor.

### 3.4. Algorithms Applying Deep Learning Architecture

Data presented in Table 2 showcase the 22 studies that applied DL algorithms [6,26,27,28,29,30,31,32,33,34,35,36,37,38,39,40,41,42,43,44,45,46,47]. Some of the authors tested different types of algorithms; the results shown in Table 2 are the best performing algorithms presented in the literature. Furthermore, if the data were unable to be obtained from the publication, this is stated as Not Available (NA).

### 3.5. Convolutional Neural Network (Twelve Studies)

The convolution neural network architecture is the most frequently applied architecture in Table 2 [6,26,27,28,29,30,31,32,33,34,35,36]. The CNN architecture reached an accuracy of 82.2%–97.6%, sensitivity of 83.1%–96.6%, specificity of 71.4%–98.2%, and an AUC of 0.87%–0.98%. Da Silva et al. increased the number of nodules and achieved superlative results. The studies included in this review that applied the PSO algorithm are all included in the top three best-achieving algorithms.

### 3.6. Deep Believe Network

Zhang et al. is the only study which applied a deep believe network [37]. The algorithm is trained to detect large nodules >30 mm, and achieved results above 90% with regards to accuracy, sensitivity and specificity. Two out of 14 articles applied a deep convolutional neural network (DCNN). The articles reached an accuracy of 89.0%–89.5% and a sensitivity of 84.2%–87.1% [39,40].

### 3.7. Other

Abbas et al. [44] chose deep neural network architecture and achieved results above 94% with regards to accuracy, sensitivity and specificity. Gruetzemacher et al. [43] also applied a DNN architecture and achieved above 94% in sensitivity. The study by Nibali et al. [46] is the only study that applied a deep residual neural network and achieved accuracy, sensitivity, and specificity above 88% [46]. Shaffie et al. [42] and Naqi et al. [47] both presented an architecture utilizing autoencoders. Naqi et al. [47] presented results above 95% in accuracy, sensitivity, and specificity. Shaffie et al. presented results above 85%. Xie et al. [38] proposed an algorithm utilizing the multiview knowledge-based collaborative (MV-KBC) deep model to separate malignant from benign nodules using limited chest CT data.

## 4. Discussion

The included studies all applied to the largest annotated image archive of CT scans of the lungs [4]. All included articles were able to detect lung nodules with a high accuracy, sensitivity, and specificity, using ML. The majority of the algorithms achieved results above 90% in one or more of the four diagnostic performing parameters. However, there is no consensus on the methods applied to determine the efficiency of ML algorithms, and the heterogeneity in the selection of included scans and the different parameters for the algorithms makes it challenging to compare them.

Applying ML algorithms to medical images comes with several limitations, one of the most profound being the lack of labeled training data. The lack of large training datasets is often mentioned as an obstacle, therefore, databases such as LIDC-IDRI are greatly appreciated and applied for training and validating algorithms. It should be noted that over the course of at least a decade, most Western hospitals have used picture archiving and communication system (PACS) systems in radiology. This magnitude of imaging data acquired for specific purposes in well-structured archives is uncommon. The main challenge is, thus, not the availability of image data itself, but the acquisition of relevant annotations/labeling for these images. Free-text reports on the radiologists’ findings are stored on the PACS system. Turning these reports into accurate labeling of structures and findings can be challenging and requires sophisticated text-mining methods, which is an important field of study in itself, where deep learning is also widely used nowadays. Introducing a structured reporting system would become very beneficial in an ML objective; this could potentially lead to improvement of radiologic findings and, eventually, patient care.

The general architecture of feature-based and DL algorithms differ. The specific architecture within the two groups also differs—the architectures and restrictions are set by the author. These are some of the contributing factors for the difference in performance when applying the same general architecture. This contributes to the difficulty in comparison. The algorithms applying feature-based architecture generated, overall, better results compared to the algorithms applying a DL approach [29,47]. The feature-based algorithms consist of different steps; the first step is usually computing the image features that will be of importance in the prediction process. The best combination of features is then selected, and the features can be applied to classify the image. The features are often based on texture, shape, or size of a nodule. The annotations given in the LIDC-IDRI dataset can be extracted, and the algorithm is able to learn to make a prediction. When the author is training the algorithm, it is possible to optimize the parameters when diagnosing correctly, thus improving the performance. The benefits of applying a DL algorithm is that the algorithm does not need feature identification as the first step. The algorithm identifies the features as a part of the learning process. The definition of DL is an algorithm which applies neural networks with multiple layers, usually more than 20. This has become possible because of the tools initially created for computer gaming and the massive parallel computing power of a graphics processing unit.

One of the most beneficial reasons for applying a DL algorithm is the learning curve. The DL algorithm is able to improve the performance over time, compared to a feature-based algorithm. The weight of the feature is set by the author and cannot be altered. The different types of DL algorithms have different abilities.

Several larger companies have evolved image recognition algorithms, and these algorithms are pre-trained on images that are not specific to the task. An example of this is Google’s GoogLeNet [48], which is trained on more than a million images from the ImageNet database. GoogLeNet has a rich feature representation and selection which can be incorporated into an algorithm made for nodule detection.

Ramachandran et al. [31] incorporated this technique into the algorithm they presented. The algorithm proposed a new object detection workflow, using a convolutional neural network to detect nodules in images and define the bounding boxes around them. The system presented an architecture based on DetectNet, which incorporates GoogLeNet inception layers without the fully connected layers.

Several of the proposed techniques have the potential for building medical diagnosis tools. Five of the feature-based and two of the DL algorithms presented an accuracy >95% [7,10,20,21,29,47]. These contributions should move forward from the LIDC-IDRI database and be taken into consideration with regard to implementing this technique for clinical practice. This could lead to an increase in nodule detection. To our knowledge, there are no studies published concerning this topic.

## 5. Conclusions

In conclusion, studies on ML and DL algorithms are able to detect lung nodules at a high level of accuracy, sensitivity, and specificity using ML when applied to an annotated archive of CT scans of the lung. However, there is no consensus on the method applied to determine the efficiency of ML algorithms. So far, there are no studies demonstrating in which clinical setting to ML could be used, and whether or not this would lead to detection of a higher number of lung nodules.

## Figures and Tables

**Table 1:** Feature-based algorithms applied to the Lung Image Database Consortium Image Collection (LIDC-IDRI) database.

Author	Year	CT Scans Incl.	Accuracy (%)	Sensitivity (%)	Specificity (%)	AUC	Classifier	Nodule Type	Selected Features
Akram et al. * [7]	2015	84	96.6	96.9	96.3	0.980	SVM	All types	2D and 3D geometric and intensity statistical features
Alilou et al. * [8]	2014	60	NA	80.0	NA	NA	SVM	Solid	2D and 3D subset of features
Bai et al. [9]	2015	99	NA	80.0	NA	NA	NA	All types	Local shape analysis and data-driven local contextual feature learning
Choi et al. * [10]	2014	84	99.0	97.5	97.5	0.998	SVM-r	All types	CAD system for different dimensions of AHSN features
El Regaily et al. [11]	2017	400	70.5	77.7	69.5	NA	The simple rule classifier	All types	Geometric and intensity statistical features
Firmino et al. * [12]	2016	420	NA	94.4	NA	NA	SVM	All types	HOG; watershed; features of texture, shape, and appearance
Gonçalves et al. * [13]	2018	NA	68.4	55.0	87.5	0.905	SVM	Solid nodules	Intensity-, texture-, and shape-based features
Gong et al. * [14]	2016	100	91.5	90.2	91.5	0.960	FLDA	Not GGO	11 selected image features
Gupta et al. [15]	2017	899	NA	90.0	NA	0.980	softmax	Large nodules	Feature mapping: stacked sparse autoencoder (SSAE)
Hancock et al. * [16]	2017	619	88.0	84.6	NA	0.949	Nonlinear	All types	Nonlinear classifier, diameter, and volume features included
Jaffar et al. [17]	2018	59	98.8	98.4	98.7	0.999	Random forest	All types	Novel ensemble shape gradient features (NESGF)
Liu et al. [18]	2017	107	NA	89.4	NA	NA	NA	All types	Geometric and statistical features
Lu et al. [19]	2015	98	NA	85.2	NA	NA	Regression tree	All types	Hybrid scheme based on 16 features
Naqi et al. * [20]	2018	250	99.0	98.6	98.2	0.990	SVM	All types	Geometric texture features descriptor (GTFD)
Shaukat et al. * [21]	2017	850	97.1	98.1	96.0	0.995	SVM-Gaussian	All types	Intensity, shape (2D and 3D), and texture features
Taşcı et al.* [22]	2015	24	92.9	NA	NA	0.883	GLMR	Juxtapleural	Seven shape- and texture-based features
Wang et al. * [23]	2018	NA	95.9	95.6	95.0	0.961	SS-ELM	All types	Haralick features and morphological features
Zhang et al. * [24]	2018	71	NA	89.3	NA	NA	SVM	Juxtavascular nodules	3D skeletonization
Zhao et al. [25]	2017	NA	91.2	NA	NA	0.970	softmax	All types	Global and local features

CAD: Computer-aided detection, AHSN: angular histograms of surface normal, HOG: Histogram of oriented Gradients, NA: not available. The studies marked with a star (“*”) presented several types of alterations to the algorithm, producing different results. These results are not presented in the table.

**Table 2:** Deep learning algorithms applied to the LIDC-IDRI database.

Author	Year	Malignant	Benign	Accuracy (%)	Sensitivity (%)	Specificity (%)	AUC	Noduli Type	Architecture
Chen et al. [26]	2018	NA	NA	NA	93.7	NA	NA	All types	CNN
Sun et al. [33]	2017	47576	41372	NA	NA	NA	0.890	All types	CNN
Wang et al. [34]	2017	NA	NA	NA	83.1	NA	NA	All types	CNN
Da Silva et al. [29]	2018	3415	8742	97.6	92.2	98.2	0.955	All types	CNN
Da silva et al. [28]	2017	1413	1830	94.75	94.7	95.1	0.940	All types	CNN
Causey et al. [6]	2018	NA	NA	94.6	94.8	94.3	0.984	All types	CNN
Ramachandran et al. [31]	2018	3300	3300	93.0	89.0	NA	NA	All types	CNN
Zhu et al. [36]	2018	450	554	90.4	NA	NA	NA	All types	CNN
Da Nóbrega et al. [27]	2018	NA	NA	88.4	85.3	NA	0.931	All types	CNN
Song et al. [32]	2017	2311	2265	84.2	84.0	84.3	0.910	All types	CNN
Han et al. [30]	2018	538	622	82.5	96.6	71.4	NA	GGO	CNN
Zhao X. et al. [35]	2018	375	368	82.2	NA	NA	0.877	All types	CNN
Zhang et al. [37]	2017	40800	32000	95.0	93.5	90.2	0.930	> 30 mm	DBN
Xie et al. [39]	2018	648	1324	89.53	84.2	92.0	0.960	All types	DCNN
Li et al. [40]	2016	40772	21720	89.0	87.1	NA	NA	All types	DCNN
Shaffie et al. [42]	2018	NA	NA	91.2	85.0	95.8	0.95	All types	Deep autoencoder
Gruetzemacher et al. [43]	2018	NA	NA	NA	94.2	NA	NA	All types	DNN
Abbas et al. [44]	2017	1300	1300	95.0	94.0	96.0	0.950	All types	DNN
Hamidian et al. [45]	2017	NA	NA	NA	80.0	NA	NA	All types	FCN + CNN
Xie et al. [38]	2018	644	1301	91.6	86.5	94.0	0.95	All types	MV-KBC
Nibali et al. [46]	2017	420	411	89.9	91.1	88.6	NA	All types	ResNet
Naqi et al. [47]	2018	NA	NA	96.9	95.6	97.0	NA	All types	SA + softmax
